# Extensive Comminuted Fracture of the Leg—Treated without Splints

**Published:** 1878-03-20

**Authors:** Z. B. Herndon

**Affiliations:** Richmond, Virginia


### EXTENSIVE COMMINUTED FRACTURE OF THE LEG—TREATED WITHOUT SPLINTS

By Z. B. Herndon, M.D., of Va.

On the 27th of April, 1870, Mr. B., aged 65, was one of the victims of the capitol disaster in this city. Besides being severely bruised in other respects, his right leg was so badly crushed, from the ankle to a point about two inches below the knee, that in lifting the limb with the hands it would bend like a bag of bran. There were several distinct and complete fractures.

The question of amputation, of course, arose, but the age of the patient and the great amount of shock, prevented surgical interference. With but little hope of benefit, every attention was given to personal comfort. The limb was placed, for the night, on a pillow, which was tied around it, and stimulants directed.

The next day, a box of bran was substituted. For some days this was continued, but the weather being warm, and frequent changes so necessary, (such change causing the patient great discomfort), the box was laid aside, and a strip of cloth about 18 inches long, and wide enough to extend from the knee to the foot, was placed under the limb, and its sides fastened by small hooks to the strips of wood, which were suspended by a rope over a pully attached to the top of the bed.

By this method the limb could be raised or lowered by the patient himself much to his comfort; and instead of having to remove, daily, heaps of bran saturated with pus, when an abscess formed, a small window was cut into the cloth, through which the abscess was punctured and injected with a solution of carbolic acid, dr. i to water oz. v. Pads, placed against projecting points, kept the limb somewhat in the proper position.

To overcome the exhausting effects of excessive drainage, nourishing food, milk punches, and the muriated tincture of iron, in ten drop doses every three hours, were given.

After some weeks a splint was applied with the hope of better results, but such was the agony of the patient that he cut it loose with his knife. Having escaped death, any sort of a limb was now considered a good compromise; and with a full knowledge of the fact that more than usual deformity would attend recovery, no additional appliance was used.

Close attention was given until the 12th of July. After this the patient began to ride out, and gradually to walk upon crutches. For several years before his death, (which occurred this spring), he walked two miles to his business with no other aid than that of his walking-cane. A word for *conservative surgery.*

RICHMOND, VA., Dec. 19, 1877.

